# The role of metalloproteases in fertilisation in the ascidian *Ciona robusta*

**DOI:** 10.1038/s41598-018-37721-1

**Published:** 2019-01-30

**Authors:** Shiori Nakazawa, Maki Shirae-Kurabayashi, Hitoshi Sawada

**Affiliations:** 1 Sugashima Marine Biological Laboratory, Graduate School of Science, Nagoya University, 429-63 Sugashima, Toba, 517-0004 Mie Japan; 2 Present Address: Hitachi, Ltd., Research & Development Group, Akanuma, Hatoyama, Hiki, Saitama Japan

## Abstract

In the ascidian *Ciona robusta* (formerly *C. intestinalis* type A), the mechanism underlying sperm penetration through the egg investment remains unknown. We previously reported that proteins containing both an astacin metalloprotease domain and thrombospondin type 1 repeats are abundant in the sperm surface protein-enriched fraction of *C. robusta*. Here we investigated the involvement of those proteins in fertilisation. We refined the sequences of astacin metalloproteases, confirmed that five of them are present in the sperm, and labelled them as tunicate astacin and thrombospondin type 1 repeat-containing (Tast) proteins. Fertilisation of *C. robusta* eggs was potently inhibited by a metalloprotease inhibitor GM6001. The eggs cleaved normally when they were vitelline coat-free or the inhibitor was added after insemination. Furthermore, vitelline coat proteins were degraded after incubation with intact sperm. These results suggest that sperm metalloproteases are indispensable for fertilisation, probably owing to direct or indirect mediation of vitelline-coat digestion during sperm penetration. TALEN-mediated knockout of *Tast* genes and the presence of GM6001 impaired larval development at the metamorphic stage, suggesting that *Tast* gene products play a key role in late development.

## Introduction

Egg fertilisation is key to achieving genetic diversity in the next generation. In most animals, the eggs are covered with an acellular investment called the egg coat, also called the vitelline coat (VC) in some marine invertebrates, including ascidians and zona pellucida in mammals. The egg investment protects the eggs from mechanical damage and interspecific crossing, in addition to serving as a barrier against the sperm of even the same species. Sperm are therefore equipped with specialised systems to recognise and penetrate the egg coat. This includes a lytic agent which acts against the egg coat, referred to as lysin.

The ascidian *Ciona robusta* (Formerly *C. intestinalis* type A; see Brunetti *et al*.^1^ and Pennati *et al*.^2^ for details; note that it is still referred to with the former name in NCBI Protein database and some publications) is a model organism for exploring the mechanisms underlying fertilisation owing to the easy acquisition of mature gametes, the established database and the 3-month life span. We have previously performed proteomic analyses of sperm-surface proteins and those released from the sperm^3^ to document the sperm proteins that would mediate gamete interactions in *C. robusta*. Proteins containing both an astacin metalloprotease domain and thrombospondin type 1 repeats were abundant in the sperm-surface protein-enriched fraction prepared by labeling sperm with a cell-impermeable biotinylating agent followed by affinity purification with avidin.

The role of sperm-based astacin metalloproteases in reproduction remains unclear, with the only exception of Semp1^4^, which is the seminal-fluid protease in *Drosophila melanogaster*. Semp1 processes the ovulation hormone ovulin and the sperm storage protein Acp36DE in the female reproductive tract^4,5^.

In the ascidian *Halocynthia roretzi*, the ubiquitin-proteasome system is thought to mediate the digestion of the VC by the sperm^6^, but its relevance in *C. robusta* remains unclear. Although the abundance of astacin metalloproteases on the sperm surface of *C. robusta* suggests that the proteins play an essential role in gamete interaction, no astacin family metalloprotease has been shown to be involved in fertilisation as a spermatic factor. Therefore, we aimed to elucidate the function of *C. robusta* metalloproteases in fertilisation.

## Results

### GM6001 affects fertilisation

**Figure 1:**
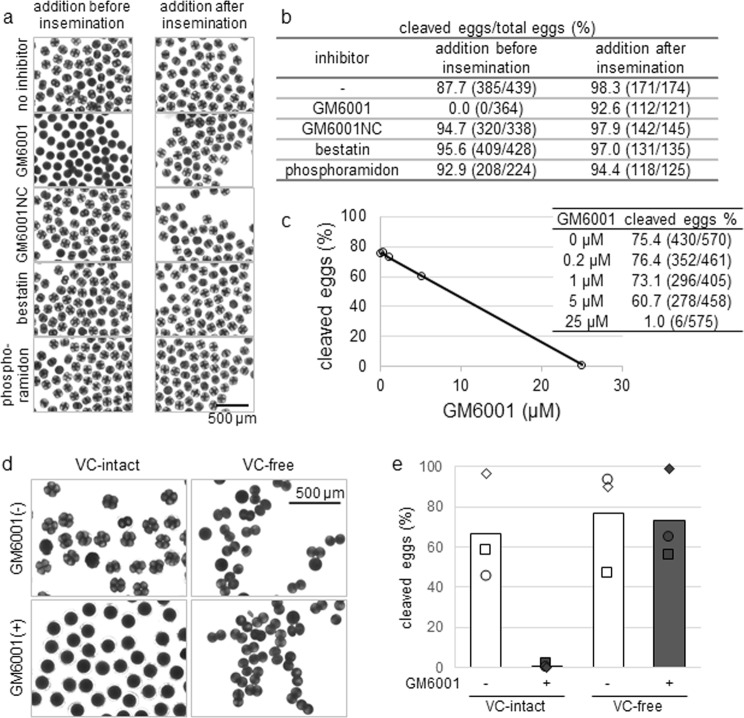
GM6001 inhibited fertilisation of *C. robusta* eggs only in the presence of VC. (**a**,**b**) Eggs were inseminated in the presence (left) and in the absence of inhibitors and were then exposed to inhibitors (right). Fertilised eggs are at the 2- or 4-cell stages. (**c**) The dose dependency of the inhibitory effect of GM6001 on the ratio of cleaving eggs. (**d**,**e**) Eggs with the VC intact or deprived were inseminated in the presence or absence of GM6001 (*N* = 3, markers indicate independent data points, bars indicate means).

Putative metalloproteases belonging to the astacin superfamily were abundant in the surface protein-enriched fraction of *C. robusta* sperm, as mentioned earlier^3^. To check the role of metalloproteases in fertilisation, *C. robusta* eggs were inseminated in the presence of the following metalloprotease inhibitors (experiments are briefly described in Fig. S1): wide-spectrum metalloprotease inhibitor GM6001, inhibitory activity-free analogue GM6001NC, aminopeptidase-selective inhibitor bestatin, and thermolysin-selective and bacterial metalloprotease-selective inhibitor phosphoramidon (Fig. 1). Most eggs (no inhibitor: 385 cleaved eggs/439 total eggs, 87.7% egg cleavage; GM6001NC: 320/338, 94.7%; bestatin: 409/428, 95.6%; phosphoramidon: 208/224, 92.9%) underwent cleavage, and few (0/364, 0.0% egg cleavage) that were inseminated in the presence of GM6001 achieved cleavage at a concentration of 25 μM (Fig. 1a,b). Most eggs exposed to inhibitors at the same concentration after insemination (no inhibitor: 171/174, 98.3%; GM6001: 112/121, 92.6%; GM6001NC: 142/145, 97.9%; bestatin: 131/135 97.0%; phosphoramidon: 118/125, 94.4%) underwent cleavage, suggesting that the decreased cleavage ratio was the result of inhibited fertilisation instead of egg cleavage itself. The inhibitory effect of GM6001 was dose dependent (0 µM: 430 cleaved eggs/570 total eggs, 75.4% cleaved eggs; 0.2 µM: 352/461, 76.4%; 1 µM: 296/405, 73.1%; 5 µM: 278/458, 60.7%; 25 µM: 6/575, 1.0%) (Fig. 1c). To clarify whether the inhibition occurred before or after sperm penetration through the VC, eggs deprived of the VC were inseminated (Fig. 1d,e). VC-free eggs were cleaved after insemination in the presence of 25 μM GM6001 (73.3% egg cleavage) and absence of it (76.9%) whereas VC-intact eggs were affected (66.9% and 0.7% egg cleavage in the absence and presence of GM6001, respectively), indicating that GM6001 inhibited fertilisation by blocking sperm penetration through the VC, or through a process before this stage.

### Sperm-surface metalloproteases are needed for VC digestion

**Figure 2:**
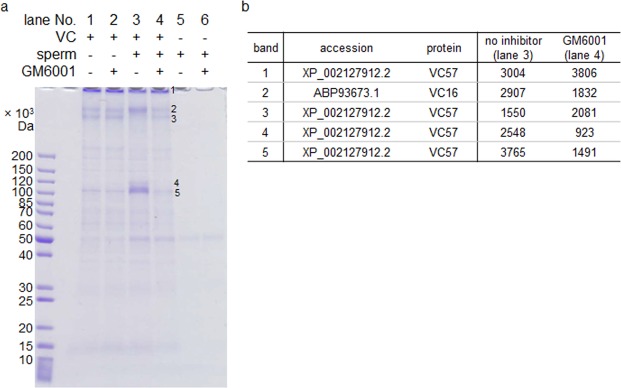
Digestion of the VC by the sperm was inhibited by GM6001. (**a**) Isolated VC and intact sperm of *C. robusta* were incubated together in the absence or presence of GM6001. Changes in the intensity caused by sperm were observed in the five labelled bands (1–5), all of which were cancelled by the addition of GM6001. These bands in lanes 3 and 4 were analysed by mass spectrometry. The full-length gel image is available in Fig. S6. (**b**) The top hit proteins detected from the bands and their protein scores (Mascot ver 2.4.1).

In order to investigate whether sperm surface GM6001-sensitive proteases (i.e. metalloproteases) are involved in digestion of the VC, VCs mechanically isolated from the *C. robusta* eggs were incubated with sperm. Incubation with sperm in the absence of the inhibitor caused changes in the band pattern of the VC (Fig. 2a), whereas these changes were inhibited by GM6001. Mass spectrometry identified the affected bands as VC57 and VC16, two of the major constituents of the *C. robusta* VC^7^ (Fig. 2b). These results suggested that sperm metalloprotease(s) are necessary for either direct digestion of the VC or activation of the VC-digesting enzyme(s).

### At least five metalloproteases are present in *C. robusta* sperm

We previously reported that the most prominent metalloproteases in the sperm surface protein-enriched fraction of *C. robusta* were astacin family metalloproteases: XP_002131733.1, XP_009857439.1, XP_002119895.1 and BAH59283.1 (NCBI Protein database)^3^. They were found to correspond with KH.C9.806, KH.C1.618, KH.C1.533 and KH.C1.493 respectively in the KH2012 gene model by BLAST search on the Aniseed database. Another putative protein with an astacin domain and thrombospondin type 1 repeats, KH.C1.332, was also found in the KH2012 gene model.

**Figure 3:**
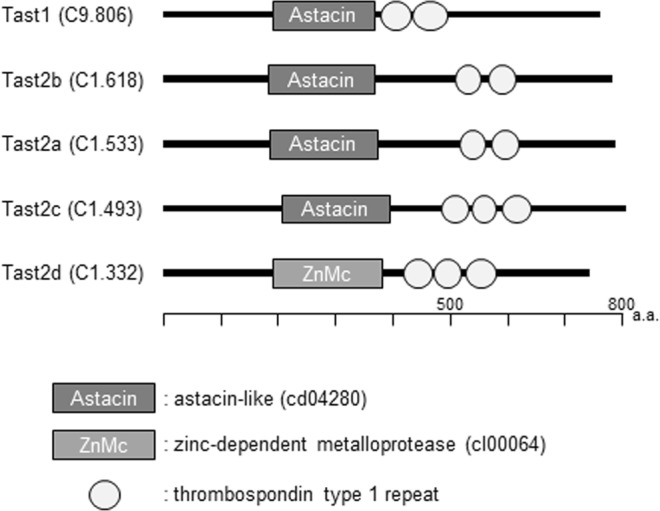
The domain compositions of *C. robusta Tast* gene products predicted by NCBI Conserved Domain Search. Detected conserved motifs with the E-values less than 1E-04 are shown.

These five proteins were cloned, and their sequences were confirmed. The refined sequences are shown in Fig. S2 and were designated in the NCBI Protein database (accession numbers: MH108630, MH108631, MH108632, MH108633 and MH108634). The predicted domain compositions based on the refined sequences (Fig. 3) showed striking similarity to each other, possessing one astacin family metalloprotease domain (except C1.332; the subtype of its zinc metalloprotease domain was not identified) followed by two or three thrombospondin type 1 repeats. No signal sequence, transmembrane region or consensus for an anchor was detected. The mass spectra acquired from the sperm-surface protein-enriched fraction^3^ were re-searched against a database consisting of these five refined sequences. All the five proteins were detected (Fig. S3) in both intact and ionomycin-treated sperm, indicating that these proteins are probably present on the surface of the *C. robusta* sperm, before and after sperm activation.

**Figure 4:**
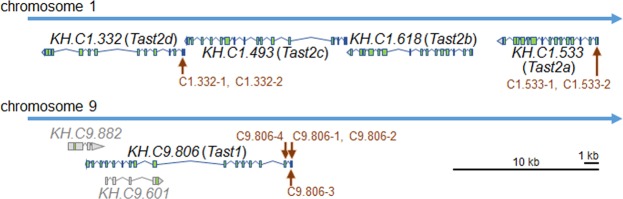
The loci of *Tast* genes in the genome of *C. robusta* (KH2012). Exons are indicated with rectangles. Introns are shown with bent lines. The predicted ORFs are highlighted green. Arrows and labels indicate the target sites and the names of the TALEN pairs (Fig. S4).

*C9.806* was coded in chromosome 9, and *C1.533*, *C1.618*, *C1.493* and *C1.332* formed a gene cluster in chromosome 1 (Fig. 4), and the predicted amino acid sequences of *C1.533, C1.618, C1.493* and *C1.332* showed higher similarity to each other than to that of *C9.806* (Fig. S2).

Proteins with the same domain composition (CBY07040.1, CBY10777.1, CBY14419.1, CBY19488.1, CBY21110.1, CBY32407.1 and CBY35258.1) were found to be present in the appendicularian *Oikopleura dioica* but not in vertebrates by using BLAST search in the NCBI Protein database. Therefore, we labelled these proteins as tunicate astacin domain and thrombospondin type 1 repeats-containing (Tast) proteins. C9.806 was labelled Tast1, and C1.533, C1.618, C1.493 and C1.332 were respectively labelled Tast2a, Tast2b, Tast2c and Tast2d according to the order in which they are encoded in the gene cluster (Fig. 4).

### Metalloproteases are essential for post-embryonic viability

**Figure 5:**
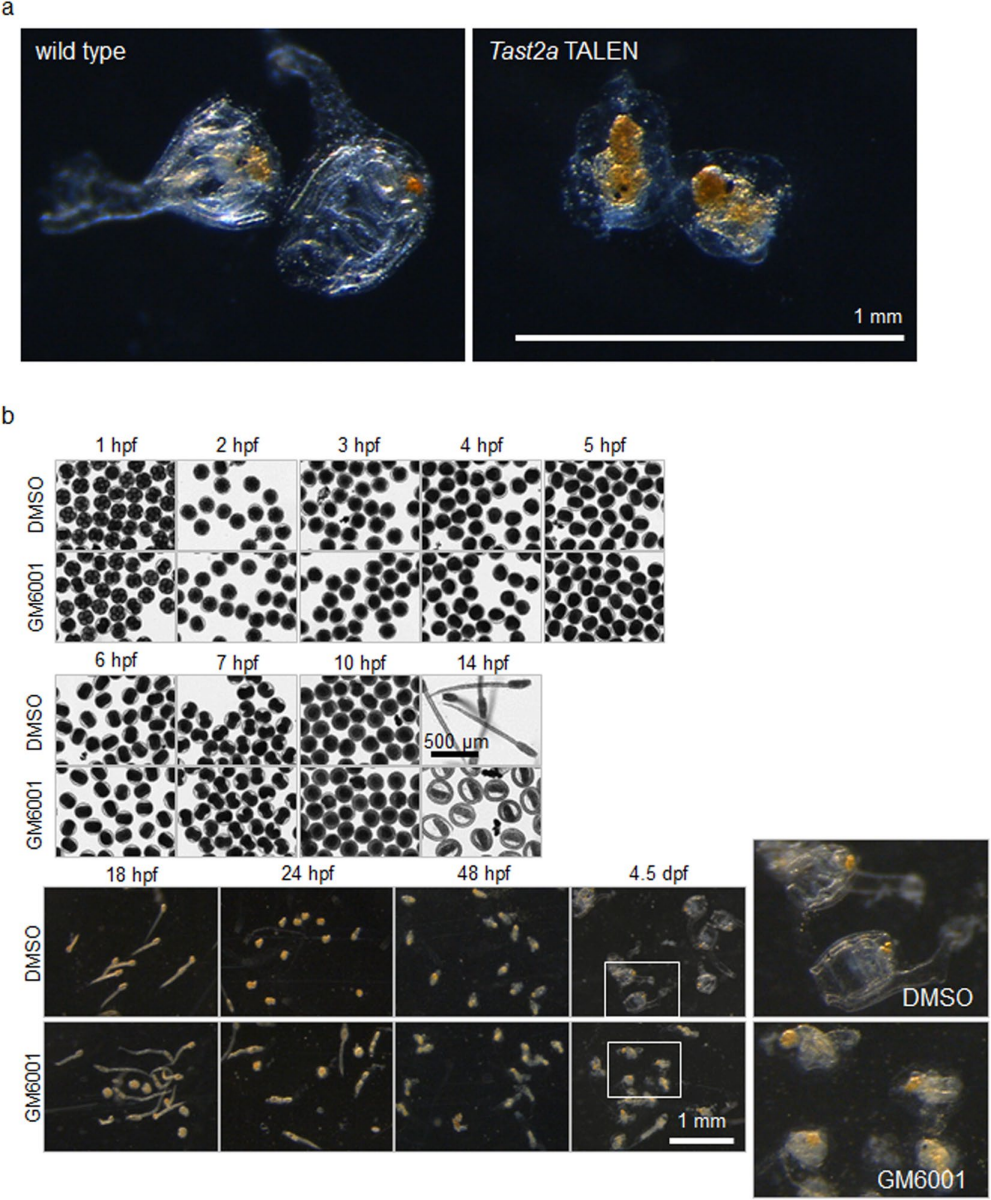
Depletion of metalloproteases caused developmental anomaly at the metamorphic stage. (**a**) Examples of 6 day juveniles injected with C1.533-1 TALEN pairs (right) and their wild type siblings (left). (**b**) Development of wild type fertilised eggs in the absence or presence of 25 μM GM6001. Embryos seemed normal until the hatching was slightly delayed in embryos exposed to GM6001. Larvae in both conditions went through tail contraction; however, only GM6001-free larvae achieved metamorphosis.

To examine the involvement of Tast proteins in fertilisation, TALEN-mediated knockout was performed. The TALEN pairs (Fig. S4) were designed to target the first or second exons of *Tast1*, *Tast2a* and *Tast2d* with a single knockout aimed for *Tast1* and a cluster-wide knockout for *Tast2a*, *Tast2b*, *Tast2c* and *Tast2d*. The TALEN pairs resulted in a high efficiency of mutagenesis (Fig. S4d–f); however, the mutated larvae went through an apparently impaired development during the metamorphic stage (Fig. 5a). In addition, the effect of GM6001 and expression of *Tast* genes during development were examined in order to address the possibility that metalloproteases, or Tasts, take part in development. Wild type fertilised eggs incubated in the presence of GM6001 showed failed metamorphosis (Fig. 5b), even though they appeared normal during the earlier stages with a slight delay in hatching out (14 hpf in Fig. 5b), slightly bent tails (18 hpf), and moderate delay in tail contraction (24 hpf). The mRNAs of *Tast1*, *Tast2a* and *Tast2d* were found to be expressed during the metamorphic stage (Fig. S5).

## Discussion

Inhibition of fertilisation (Fig. 1) by GM6001 but not by bestatin or phosphoramidon suggests that metalloprotease(s) belong to bestatin- or phosphoramidon-insensitive and GM6001-sensitive subtype(s) would be required for fertilisation.

The successful fertilisation of VC-free eggs even in the presence of GM6001 (Fig. 1d,e) suggests that the target(s) of GM6001 in fertilisation would be involved in event(s) before the sperm enter the perivitelline space, i.e. when the sperm bind to the VC or when they penetrate the VC.

Both scenarios are plausible–inhibitors of aminopeptidase B (*o*-phanenthroline, bestatin and arphamenine B) have been shown to attenuate sperm binding to the vitelline envelope in *Xenopus laevis*^8^. Murine ADAMTS10, a disintegrin and metalloprotease with thrombospondin motif 10, participates in sperm adhesion to the zona pellucida^9^. These studies confirm that metalloproteases on sperm serve as the receptor for the egg coat. Moreover, sea urchin EBR1, the surface receptor for sperm expressed on the egg, also contains an ADAMTS domain^10^. It is also significant that mouse ovastacin is an astacin family metalloprotease released from cortical granules and responsible for the post-fertilisation cleavage of ZP2^11,12^, which inhibits the sperm from binding to 2-cell embryos. Hatching enzymes, astacin family metalloproteases secreted from the hatching gland cells of the embryos of ray-finned fishes and from amphibians, are known to digest the egg envelopes^13^. These studies indicate that astacin metalloproteases play the role of lytic agents, digesting the egg envelopes for the sperm penetration.

Intact sperm of *C. robusta* altered the SDS-PAGE band pattern of VC57, the main VC component; these changes in the band pattern were inhibited by GM6001 (Fig. 2). This suggests that the sperm metalloprotease(s) either directly serve as the lysin(s) or are necessary for the activation of true lysin(s). Whether the metalloproteases responsible for digestion of the VC are Tast proteins or other undetected metalloproteases is still unclear. Tasts are the most promising candidates for GM6001-mediated inhibition of fertilisation and digestion of the VC, given that most of the metalloproteases detected in the sperm-surface protein-enriched fraction^3^ were Tasts despite at least 18 non-astacin metzincin genes being present in the *C. robusta* genome^14^. However, the possibility of undetected proteases cannot be ruled out.

Previous studies have reported that metalloproteases are involved in sperm–egg fusion in *Ciona* spp.^15^ and sea urhcins^16^, along with playing a part in the acrosome reaction in sea urchins^17^. In these studies, metal chelators and/or a succinyl-Ala-Ala-Phe-MCA substrates were tested. These inhibitors are not specific to metalloproteases, and their IC_50_ values were as high as 0.5–1 mM. Although our finding that VC-free eggs were fertilised in the presence of GM6001 did not support the involvement of metalloproteases in gamete fusion, it is still possible that GM6001-resistant metalloproteases mediate gamete fusion.

The interference with the late development by TALEN-mediated knockout of *Tast* genes or GM6001 suggests that metalloproteases, possibly Tasts, also play important roles after fertilization (Fig. 5). Expression of at least three of the *Tast* genes during the metamorphic stage (Fig. S5) supports this hypothesis. Little is known about the involvement of astacin-like metalloproteases in the development of ascidians. *No va*, a gene encoding an astacin family protein is expressed maternally and zygotically in the mesenchyme of neurula- and tailbud-stage embryos in *C. intestinalis*^18^. The ascidian ortholog of *Tolloid* is thought to be involved in the regulation of cell migration^19^. In vertebrates, metalloproteases play an indispensable role in development; e.g. BMP-1 and tolloid-like protein are involved in dorsal–ventral patterning and skeletogenesis^20^, and matrix metalloproteinases play a role in skeletogenesis^21,22^. Thus, Tasts and other metalloproteases could be involved in the development of ascidians.

The lethality caused by the knockout of even one of the five *Tast* genes suggests that either these genes are functionally differentiated and incapable of compensating each other, or that the total amount of the products of these five genes is critical for normal development. This explains why multiple copies of the similar genes are encoded in the genome of *C. robusta*. Germ line-selective knockout technique^23^ will be required to avoid the lethal effect of Tast depletion and to analyse their function in fertilisation.

Metalloproteases of *Ciona* spp. may also be involved in hatching considering the delayed hatching in the presence of GM6001 (Fig. 5b) and the role of metalloprotease in the hatching of *C. intestinalis*^24^, as expressed at the gastrula stage in the ectoderm^25^. The relevance of Tasts and the ascidian hatching enzyme, however, should be further examined because the reported N-terminus sequence of the ascidian hatching enzyme (MLNPGSILTLFLMILAGTQH)^24^ currently shows no BLAST hit in the gene model of *C. robusta* (KH2012) and the genome assemblies of *C. robusta* (KH2012) and *C. intestinalis* (MTP2012).

## Conclusions

Metalloproteases are required for sperm penetration through the VC or steps before that in fertilisation of the ascidian *C. robusta*. A metalloprotease inhibitor interfered with digestion of the VC by the sperm, indicating that sperm metalloprotease(s) should either directly cleave the components of the VC or mediate activation of the true lysin(s). Tasts, the astacin metalloproteases with thrombospondin type 1 repeats, are the top candidates for the responsible metalloproteases, given their abundance on the sperm surface. Furthermore, we found that metalloproteases are also needed later in development. The proteolytic activity, the substrates and the activator of Tasts need further study. Genetic approaches including conditional knockout will help elucidation of the functions of *Tast* genes as well. A limited taxonomic range of animals, i.e., tunicates, seem to have *Tast* genes on their genomes. It would also be interesting to compare Tasts with the functional counterparts of other chordates (vertebrates and cephalochordates) if any, or to address the evolutionary origin of Tasts in regard to their uniqueness in tunicates after through elucidation of the functions of Tasts.

## Materials and Methods

### Animals

*C. robusta* was purchased from the National BioResource Project of MEXT (Japan).

### Cloning and sequencing of *Tast* genes

Testis cDNA of *C. robusta* was prepared using SuperScript III First-Strand Synthesis System (18080051; Thermo Fisher Scientific, Waltham, MA, USA) with an oligo (dT)_20_ primer by transcribing 0.5 μg of mRNA prepared from the testes of six adults using RNAzol RT (RN 190; Molecular Research Center, Inc., Cincinnati, OH, USA) following the manufacturer instructions. Tast cDNAs were amplified using *Pfu* DNA polymerase (M7741; Promega, Madison, WI, USA). The primers (Supplementary Table 1) were designed on the basis of sequences designated as C9.806.v1.A.nonSL4-1, C1.618.v1.A.SL2-1, C1.533.v1.A.ND1-1, C1.493.v1.A.SL1-1 and C1.332.v1.A.SL1-1 in the KH 2012 transcript model of *C. robusta* acquired from the Aniseed database (https://www.aniseed.cnrs.fr/) and cloned into pBluescript SK(+) vector. 5′-RACE was performed to determine the 5′ terminal sequence of *C1.493* as the amino acid sequence of C1.493.v1.A.SL1-1 on the gene model was shorter at the N-terminus by about 100 residues than those of other *Tast* genes. The testis cDNA was modified with an oligo (dG) overhang using terminal deoxynucleotidyl transferase (2230 A; Takara Bio Inc., Shiga, Japan) and was amplified with PCR by using the primers shown in Supplementary Table 2. The 5′-RACE product was cloned using TOPO TA Cloning Kit for Sequencing (450071; Thermo Fisher Scientific). The sequences of the cloned constructs were analysed using BigDye Terminator v3.1 Cycle Sequencing Kit (4337455; Thermo Fisher Scientific); the primers are shown in Supplementary Table 3.

### Fertilisation assay

Procedures for gamete collection and fertilization assay are illustrated in Fig. S1a–c. The sperm were surgically harvested from the sperm duct with a micropipette. The eggs were surgically harvested from the oviduct. After transferring them to a glass dish, they were defolliculated by passing them through a glass pipette in filtered seawater (FSW), and washing them in it.

The suspension of defolliculated eggs in FSW was aliquoted into a 12- or 24-well dish, and they were inseminated by adding 5–15 μL of 1000- or 2000-fold diluted suspension of sperm.

The inhibitors were used at a final concentration of 25 μM, unless otherwise mentioned. Each sample, including the inhibitor-free control in the inhibition experiments, was loaded with dimethyl sulfoxide at a final concentration of 0.1% (v/v).

VC-free eggs were prepared by suspending the surgically collected eggs in 2% sodium thioglycolate and 0.1% actinase E (90002-1611; Kaken Pharmaceutical, Tokyo, Japan) in FSW and then by adding 1/20-fold volume of 1 M sodium hydroxide to the suspension, pipetting the mixture at room temperature and washing the eggs with FSW.

### TALEN-mediated knockout

Plasmids coding TALE nucleases were constructed using the Platinum Gate TALEN Kit (1000000043: Addgene, Cambridge, MA, USA)^26^. mRNAs coding the TALE nucleases were synthesised using mMESSAGE mMACHINE T7 Transcription kit (AM1344; Thermo Fisher Scientific) and injected into dechorionated unfertilised eggs with a glass capillary at the concentration of 150 ng/μL each with rhodamine dextran as an injection marker (Fig. S1e).

The mutagenic efficiency of TALEN pairs was examined by amplifying the target regions from the genomic DNA extracted from a mixture of 10 tailbud larvae with primers shown in Supplementary Table 4, cloning the products with TOPO TA Cloning Kit (450641; Thermo Fisher Scientific), performing colony PCR with M13For and M13Rev primers, and analysing the sequence of the gel exudates after agarose gel electrophoresis using BigDye Terminator v3.1 Cycle Sequencing Kit.

### Digestion assay

Experimental procedures are schematically shown in Fig. S1d. The VC of *C. robusta* was isolated by repeatedly squeezing defolliculated eggs with a plastic pestle in a 1.5 mL tube, and then passing the eggs through a 26-gauge injection needle in 0.2 × calcium- and magnesium-free artificial seawater (0.2 × CM-free ASW; 86 mM NaCl, 2 mM KCl, 4 mM EPPS, pH 8.0) supplied with a protease inhibitor cocktail (cOmplete mini EDTA-free, 11836170001; Merck KGaA, Darmstadt, Germany). They were then washed in 0.2 × CM-free ASW containing 0.005% Triton X-100 on a 40 μm nylon cell strainer (352340; Corning, Corning, NY, USA) and equilibrated with FSW.

Isolated VC, a 1/200-dilute of intact sperm and 25 μM GM6001 were mixed and incubated at 4 °C in 100 μL FSW overnight. The FSW-insoluble components were solubilised with 5 × SDS sample buffer (50% glycerol, 10% SDS, 0.5% bromophenol blue, 0.25 M Tris–HCl pH 6.8, supplied with 1/20-fold volume of 2-mercaptoethanol), diluted with 4-fold volume water and separated using SDS-PAGE.

### Identification of the VC proteins

The selected protein bands were excised and analysed using mass spectrometry and database search as previously described^3^ against a database consisting of the sequences of taxonomy ID 7719 (although they are labelled *C. intestinalis*, most of the entries are the sequences of *C. robusta*) acquired from NCBI Protein database on 22 June 2017, using Mascot ver 2.4.1 software (Matrix Science Inc., Boston, MA, USA).

## Supplementary information

Fig. S1, Fig. S2, Fig. S3, Fig. S4, Fig. S5, Fig. S6, Supplementary table 1, Supplementary table 2, Supplementary table 3, Supplementary table 4

## Data Availability

The raw mass spectra and the spectra converted to mascot generic format for database search will be available on Dryad (10.5061/dryad.3kc345s). The sequences of Tast proteins will be available on the NCBI Protein database (accession numbers: MH108630, MH108631, MH108632, MH108633 and MH108634).

## References

[1] Brunetti R (2015). Morphological evidence that the molecularly determined Ciona intestinalis type A and type B are different species: *Ciona robusta* and *Ciona intestinalis*. J Zool Syst Evol Res.

[2] Pennati R (2015). Morphological Differences between Larvae of the *Ciona intestinalis* Species Complex: Hints for a Valid Taxonomic Definition of Distinct Species. PloS one.

[3] Nakazawa S, Shirae-Kurabayashi M, Otsuka K, Sawada H (2015). Proteomics of ionomycin-induced ascidian sperm reaction: Released and exposed sperm proteins in the ascidian *Ciona intestinalis*. Proteomics.

[4] Ravi Ram K, Sirot LK, Wolfner MF (2006). Predicted seminal astacin-like protease is required for processing of reproductive proteins in *Drosophila melanogaster*. Proc. Natl. Acad. Sci. USA.

[5] LaFlamme BA, Ram KR, Wolfner MF (2012). The *Drosophila melanogaster* seminal fluid protease ‘seminase’ regulates proteolytic and post-mating reproductive processes. PLoS Genet..

[6] Sawada H, Mino M, Akasaka M (2014). Sperm proteases and extracellular ubiquitin-proteasome system involved in fertilization of ascidians and sea urchins. Adv. Exp. Med. Biol..

[7] Yamada L, Saito T, Taniguchi H, Sawada H, Harada Y (2009). Comprehensive egg coat proteome of the ascidian *Ciona intestinalis* reveals gamete recognition molecules involved in self-sterility. J. Biol. Chem..

[8] Kubo H, Kotani M, Yamamoto Y, Hazato T (2008). Involvement of Sperm Proteases in the Binding of Sperm to the Vitelline Envelope in Xenopus laevis. Zool Sci.

[9] Dun MD (2012). Investigation of the expression and functional significance of the novel mouse sperm protein, a disintegrin and metalloprotease with thrombospondin type 1 motifs number 10 (ADAMTS10). Int J Androl.

[10] Kamei N, Glabe C (2003). The species-specific egg receptor for sea urchin sperm adhesion is EBR1,a novel ADAMTS protein. Genes Dev.

[11] Burkart A, Xiong B, Baibakov B, Jiménez-Movilla M, Dean J (2012). Ovastacin, a cortical granule protease, cleaves ZP2 in the zona pellucida to prevent polyspermy. J Cell Biology.

[12] Avella MA, Xiong B, Dean J (2013). The molecular basis of gamete recognition in mice and humans. Mol. Hum. Reprod..

[13] Nagasawa T (2016). Evolutionary Changes in the Developmental Origin of Hatching Gland Cells in Basal Ray-Finned Fishes. Zool. Sci..

[14] Huxley-Jones J (2007). The evolution of the vertebrate metzincins; insights from *Ciona intestinalis* and *Danio rerio*. BMC Evol. Biol..

[15] De Santis R (1992). Evidence that metalloendoproteases are involved in gamete fusion of *Ciona intestinalis*, ascidia. Dev. Biol..

[16] Roe JL, Farach HA, Strittmatter WJ, Lennarz WJ (1988). Evidence for involvement of metalloendoproteases in a step in sea urchin gamete fusion. J. Cell Biol..

[17] Farach HA, Mundy DI, Strittmatter WJ, Lennarz WJ (1987). Evidence for the involvement of metalloendoproteases in the acrosome reaction in sea urchin sperm. J. Biol. Chem..

[18] Davis SW, Smith WC (2002). Expression cloning in ascidians: isolation of a novel member of the asctacin protease family. Dev. Genes Evol..

[19] Christiaen L, Stolfi A, Levine M (2010). BMP signaling coordinates gene expression and cell migration during precardiac mesoderm development. Dev Biol.

[20] Hopkins D, Keles S, Greenspan D (2007). The bone morphogenetic protein 1/Tolloid-like metalloproteinases. Matrix Biol.

[21] Vu T, Werb Z (2000). Matrix metalloproteinases: effectors of development and normal physiology. Genes Dev.

[22] Lemaître V, D’Armiento J (2006). Matrix metalloproteinases in development and disease. Birth Defects Res. C Embryo Today.

[23] Yoshida K (2017). Germ cell regeneration-mediated, enhanced mutagenesis in the ascidian *Ciona intestinalis* reveals flexible germ cell formation from different somatic cells. Dev Biol.

[24] D’Aniello A, Vincentiis M, de, Fiore MM, Di, Scippa S (1997). Hatching enzyme from the sea-squirt *Ciona intestinalis*: purification and properties. Biochim. Biophys. Acta.

[25] Scippa S, De Candia A, De, Groppelli S, De Vincentiis M (2006). Hatching enzyme immunolocalization during embryonic development of the ascidians *Ciona intestinalis* and Phallusia mammillata. Invertebr. Reprod. Dev..

[26] Sakuma T (2013). Repeating pattern of non-RVD variations in DNA-binding modules enhances TALEN activity. Sci Rep.

