# Supplementary material for: The role of metalloproteases in fertilisation in the ascidian Ciona robusta
Source: Sci Rep. 2019 Jan 30;9:1009. doi: 10.1038/s41598-018-37721-1 (PMC6353882; doi:10.1038/s41598-018-37721-1)
Supplement: Supplementary file 1 — Fig. S1, Fig. S2, Fig. S3, Fig. S4, Fig. S5, Fig. S6, Supplementary table 1, Supplementary table 2, Supplementary table 3, Supplementary table 4 [file 41598_2018_37721_MOESM1_ESM.pdf]

Supplementary Materials

Title:

The role of metalloproteases in fertilisation in the ascidian *Ciona robusta*

Authors:

Shiori Nakazawa*†, Maki Shirae-Kurabayashi, and Hitoshi Sawada*

affiliation: Sugashima Marine Biological Laboratory, Graduate School of Science, Nagoya University, 429-63 Sugashima, Toba, Mie 517-0004, Japan

*Correspondence to: Shiori Nakazawa; e-mail: s-nakazawa@nagoya-u.jp, or Hitoshi Sawada; e-mail: hsawada@bio.nagoya-u.ac.jp; Sugashima Marine Biological Laboratory, Graduate School of Science, Nagoya University, 429-63 Sugashima, Toba, Mie 517-0004, Japan

† Current affiliation: Hitachi, Ltd. Research & Development Group

Fig. S1

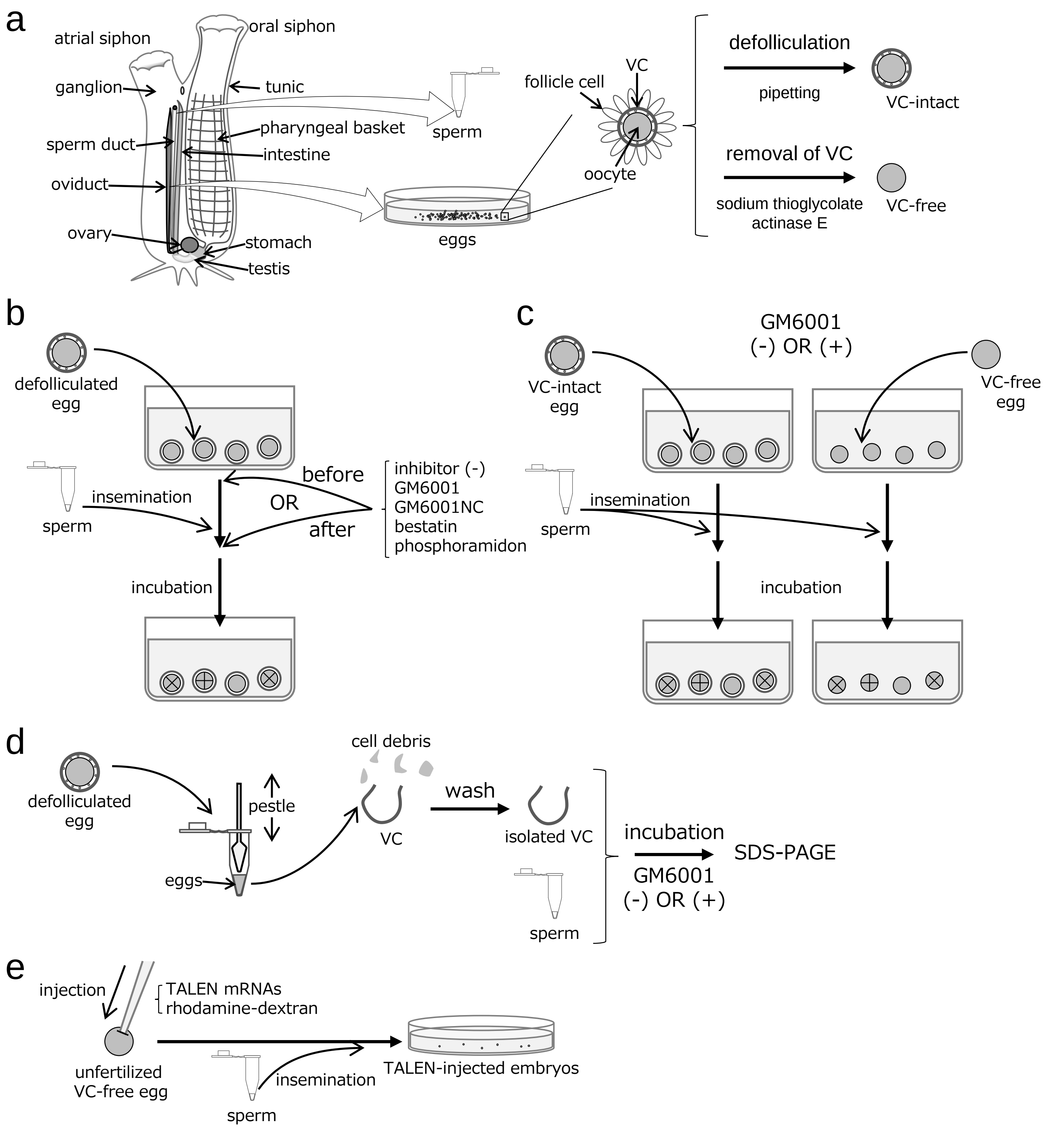

Fig. S1
Brief descriptions of experimental schemes.
a. Surgical harvest of gametes from *Ciona robusta*, defolliculation and VC-removal of the eggs.
b. Fertilization assay of VC-intact eggs in the presence or absence of inhibitors in figure 1 a-c.
c. Fertilization assay of VC-intact or -free eggs in figure 1 d-e.
d. Isolation of the VC and the digestion assay in figure 2. Eggs were mechanically crushed.
e. TALEN-mediated knockout in figure 5. Unfertilized defolliculated eggs were injected with TALEN mRNAs and then inseminated.

Fig. S2

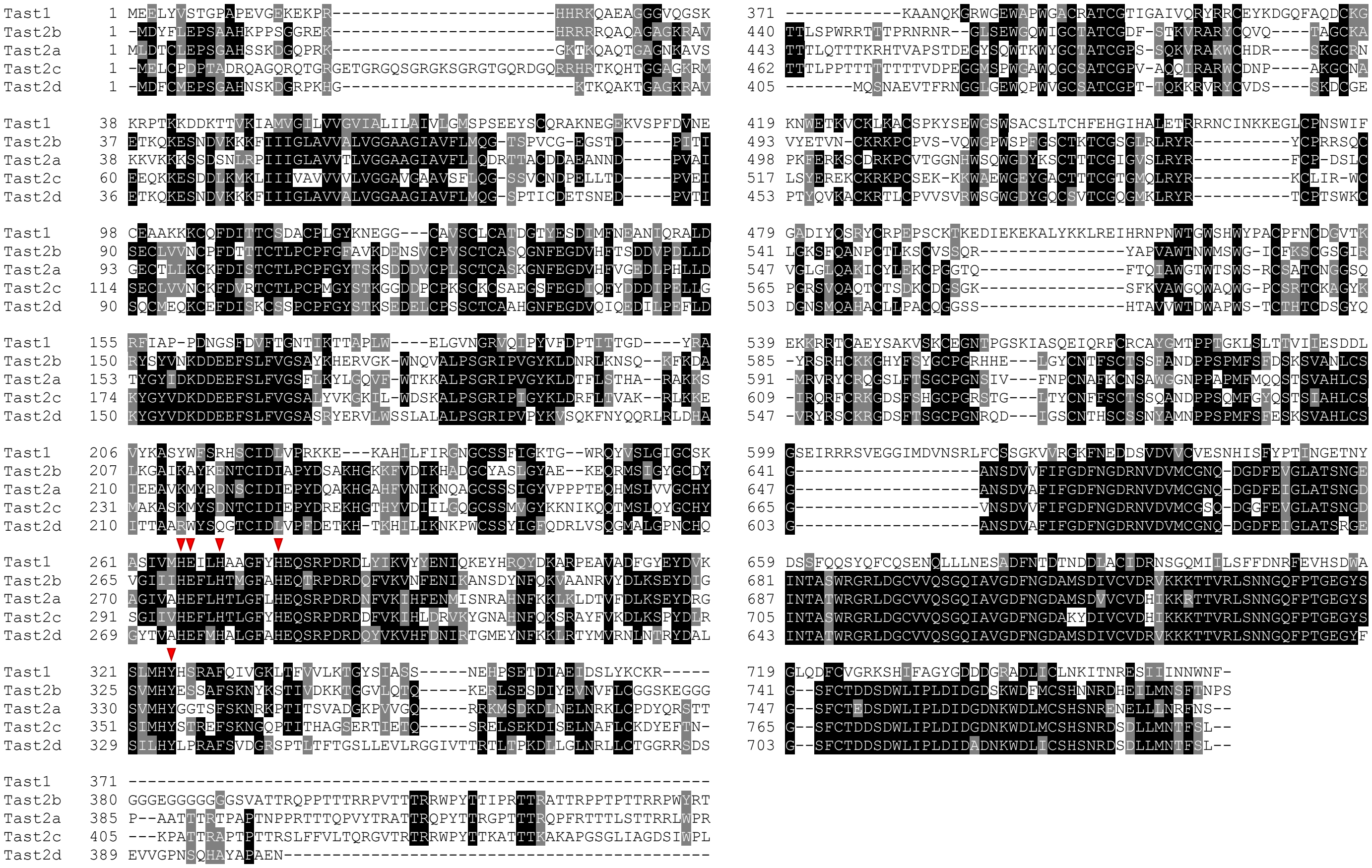

Fig. S2

Multiple sequence alignment of the refined sequences of the predicted products of *Tast* genes.
Alignment was conducted with a CLUSTALW program (http://www.genome.jp/tools-bin/clustalw) at the default settings with a sorting option -outorder=input. Arrowheads indicate the five catalytic residues of astacins (NCBI Conserved Domains cd04280).

Fig. S3

a

>C9.806_Tast1
MEELYVSTGPAPEVGEKEKPRHHRKQAEAGGGVQGSKKRPTKKDDKTTV 50
KIAMVGILVVGVIALILAIVLGMSPSEEYSCQRAKNEGEK**VSPFDVNEC** 100
EAAKK**KCQFDITTCSDACPLGYK**NEGGCAVSCLCATDGTYESDIMFNEA 150
NIQRALDRFIAPPDNGSFDVFTGNTIK**TTAPLWELGVNGRVQIPYVFDP** 200
TITTGDYRAVYK**ASYWFSRHSCIDLVPR**KKEKAHILFIR**GNGCSSFIGK** 250
TGWR**QYVSLGIGCSK**ASIVMHEILHAAGFYHEQSRPDR**DLYIKVYYENI** 300
QKEYHRQYDK**ARPEAVADFGYEYDVK**SLMHYHSR**AFQIVGK**LTFVVLK**T** 350
GYSIASSNEHPSETDIAEIDSLYKCKRKAANQKGRWGEWAPWGACR**ATC** 400
GTIGAIVQRYRRCEYKDGQFAQDCKGKNWETKVCKLKACSPKYSEWGSW 450
SACSLTCHFEHGIHALETRRRNCINKK**EGLCPNSWIFGADIYQSRYCRP** 500
EPSCKTKEDIEKEKALYKKLREIHRNPNWTGWSHWYPACPFNCDGVTKE 550
KKRRTCAEYSAKVSKCEGNTPGSK**IASQEIQR**FCRCAYGMTPPTGKLSL 600
TTVIIESDDLGSEIRRRSVEGGIMDVNSRLFCSSGKVVRGKFNEDDSVD 650
VVCVESNHISFYPTINGETNYDSSFQQSYQFCQSENQLLLNESADFNTD 700
TNDDLACIDRNSGQMIILSFFDNRFEVHSDWAGLQDFCVGRKSHIFAGY 750
GDDDGRADLICLNKITNRESIIINNWNF

b

>C1.618_Tast2b
MDYFLEPSAAHKPPSGGREKHRRRRQAQAGAGKRAVETKQKESNDVKKK 50
FIIIGLAVVALVGGAAGIAVFLMQGTSPVCGEGSTDPITISECLVVNCP 100
FDTTTCTLPCPFGFAVKDENSVCPVSCTCASQGNFEGDVHFTSDDVPDL 150
LDRYSYVNKDDEEFSLFVGSAYKHERVGK**WNQVALPSGR**IPVGYKLDNR 200
LK**NSQKFK**DALKGAIKAYK**ENTCIDIAPYDSAK**HGKK**FVDIKHADGCYA** 250
SLGYAEKEQRMSIGYGCDYVGIIIHEFLHTMGFAHEQTRPDRDQFVKVN 300
FENIK**ANSDYNFQK**VAANRVYDLKSEYDIGSVMHYESSAFSKNYKSTIV 350
DKKTGGVLQTQKER**LSESDIYEVNVFLCGGSK**EGGGGGGEGGGGGGGSV 400
ATTRQPPTTTRRPVTTTRRWPYTTIPRTTRATTRPPTPTTRRPWYRTTT 450
LSPWRRTTTPRNRNRGLSEWGQWIGCTATCGDFSTKVRAR**YCQVQTAGC** 500
KAVYETVNCKRKPCPVSVQWGPWSPFGSCTKTCGSGLRLRYRYCPRRSQ 550
CLGK**SFQANPCTLK**SCVSSQRYAPVAWTNWMSWGICFKSCGSGIRYRSR 600
HCK**KGHYFSYGCPGR**HHELGYCNTFSCTSSFANDPPSPMFSFDSKSVAN 650
LCSGANSDVVFIFGDFNGDRNVDVMCGNQDGDFEVGLATSNGEINTASW 700
RGRLDGCVVQSGQIAVGDFNGDAMSDIVCVDRVKKKTTVRLSNNGQFPT 750
GEGYSGSFCTDDSDWLIPLDIDGDSKWDFMCSHNNRDHEILMNSFTNPS 800

c

>C1.533_Tast2a
MLDTCLEPSGAHSSKDGQPRKGKTKQAQTGAGNKAVSKKVKKKSSDSNL 50
RPIIIGLAVVTLVGGAAGIAVFLLQDRTTACDDAEANNDPVAIGECTLL 100
KCK**FDISTCTLPCPFGYTSK**SDDDVCPLSCTCASKGNFEGDVHFVGEDL 150
PHLLDTYGYIDKDDEEFSLFVGSFLK**YLGQVFWTK**KALPSGRIPVGYK**L** 200
DTFLSTHARAKKSIEEAVKMYR**DNSCIDIEPYDQAKHGAHFVNIK**NQAG 250
CSSSIGYVPPPTEQHMSLVVGCHYAGIVAHEFLHTLGFLHEQSRPDRDN 300
FVKIHFENMISNRAHNFKKLKLDTVFDLKSEYDRGSVMHYGGTSFSKNR 350
KPTITSVADGKPVVGQRRKMSDKDLNELNR**KLCPDYQR**STTPAATTTRT 400
PAPTNPPRTTTQPVYTRATTRQPYTTRGPTTTRQPFRTTTLSTTRRLWP 450
RTTTLQTTTKRHTVAPSTDEGYSQWTKWYGCTATCGPSSQKVRAKWCHD 500
RSKGCRNPKFERKSCDRKPCVTGGNHWSQWGDYKSCTTTCGIGVSLRYR 550
FCPDSLCVGLGLQAKICYLEKCPGGTQFTQIAWGTWTSWSRCSATCNGG 600
SQMRVRYCRQGSLFTSGCPGNSIVFNPCNAFKCNSAWGGNPPAPMFMQQ 650
STSVAHLCSGANSDVAFIFGDFNGDRNVDVMCGNQDGDFEIGLATSNGD 700
INTATWRGRLDGCVVQSGQIAVGDFNGDAMSDVVCVDHIKKRTTVRLSN 750
NGQFPTGEGYSGSFCTEDSDWLIPLDIDGDNKWDLMCSHSNR**ENELLLN** 800
RFNS

d

>C1.493_Tast2c
MELCPDPTADRQAGQRQTGRGETGR**GQSGRGKSGR**GTGQRDGQRRHRTK 50
QHTGGAGKRMEEQKKESDDLKMKLIIIVAVVVVLVGGAVGAAVSFLQGS 100
SVCNDPELLTDPVEISECLVVNCKFDVRTCTLPCPMGYSTKGGDDPCPK 150
SCKCSAEGSFEGDIQFYDDDIPELLGKYGYVDKDDEEFSLFVGSALYVK 200
GKILWDSKALPSGR**IPIGYK**LDRFLTVAKRLKKEMAKASKMYSDNTCID 250
IEPYDREKHGTHYVDIILGQGCSSMVGYKKNIKQQTMSLQYGCHYSGII 300
VHEFLHTLGFYHEQSRPDRDDFVKIHLDRVKYGNAHNFQKSRAYFVKDL 350
KSPYDLRSIMHYSTREFSK**NGQPTITHAGSER**TIETQSRELSEK**DISEL** 400
NAFLCKDYEFTNKPATTRAPTPTTRSLFFVLTQRGVTRTRRWPYTTKAT 450
TTKAKAPGSGLIAGDSIWPLTTTLPPTTTTTTTVDPEGGMSPWGAWQG 500
CSATCGPVAQQIRARWCDNPAKGCNALSYEREKCKRKPCSEKKKWAEWG 550
EYGACTTTCGTGMQLRYRKCLIRWCPGRSVQAQTCTSDKCDGSGKSFKV 600
AWGQWAQWGPCSRTCKAGYKIRQRFCRKGDSFSHGCPGRSTGLTYCNFF 650
SCTSSQANDPPSQMFGYQSTSIAHLCSGVNSDVVFIFGDFNGDRNVDVM 700
CGSQDGGFEVGLATSNGDINTASWRGRLDGCVVQSGQIAVGDFNGDAK**Y** 750
DIVCVDHIKKKTTVRLSNNGQFPTGEGYSGSFCTDDSDWLIPLDIDGDN 800
KWDLMCSHSNRDSDLLMNTFSL

e

>C1.332_Tast2d
MDFCMEPSGAHNSKDGRPKHGKTKQAKTGAGKRAVETKQKESNDVKKKF 50
IIIGLAVVALVGGAAGIAVFLMQGSPTICDETSNEDPVTISQCMEQKCE 100
FDISKCSSPCPFGYSTKSEDELCPSSCTCAAHGNFEGDVQIQEDILPEF 150
LDKYGYVDKDDEEFSLFVGSASRYERVLWSSLALALPSGRIPVPYKVSQ 200
KFNYQQRLR**LDHAITTAAR**WYSQGTCIDLVPFDETKHTKHILIKNKPWC 250
SSYIGFQDRLVSQGMALGPNCHQGYTVAHEFMHALGFAHEQSRPDRDQY 300
VK**VHFDNIR**TGMEYNFKKLRTYMVRNLNTR**YDALSILHYLPR**AFSVDGR 350
SPTLTFTGSLLEVLRGGIVTTRTLTPKDLLGLNRLLCTGGRRSDSEVVG 400
PNSQHAYAPAENMQSNAEVTFRNGGLGEWQPWVGCSATCGPTTQKKRVR 450
YCVDSSKDCGEPTYQVK**ACKRTLCPVVSVR**WSGWGDYGQCSVTCGQGMK 500
LRYRTCPTSWKCDGNSMQAHACLLPACQGGSSHTAVVWTDWAPWSTCTH 550
TCDSGYQVRYRSCK**RGDSFTSGCPGNR**QDIGSCNTHSCSSNYAMNPPSP 600
MFSFESKSVAHLCSGANSDVAFIFGDFNGDRNVDVMCGNQDGDFEIGLA 650
TSR**GEINTATWR**GRLDGCVVQSGQIAVGDFNGDAMSDIVCVDRVKKKTT 700
VRLSNNGQFPTGEGYFGSFCTDDSDWLIPLDIDADNKWDLICSHSNRDS 750
DLLMNTFSL

f

sum of protein scores (Mascot ver 2.4.1)

	Tast1	Tast2b	Tast2a	Tast2c	Tast2d
ionomycin-	1875	832	324	318	323
ionomycin+	2506	707	581	321	425

Fig. S3 Continued

g

Tast1 188-204 VQIPYVFDPTITTGDYR

m/z 992.82, *z* = +2

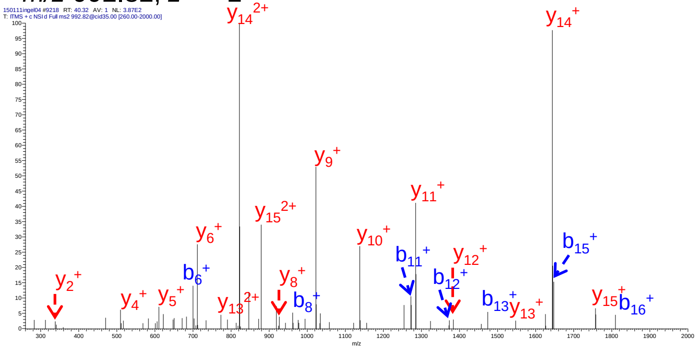

h

Tast2b 358-375 LSESDIYEVNVFLCGGSK

m/z 1009.02, *z* = +2

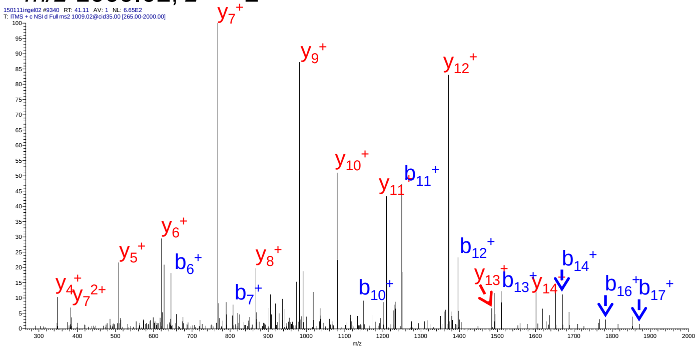

i

Tast2a 196-205 LDTFLSTHAR

m/z 580.76, *z* = +2

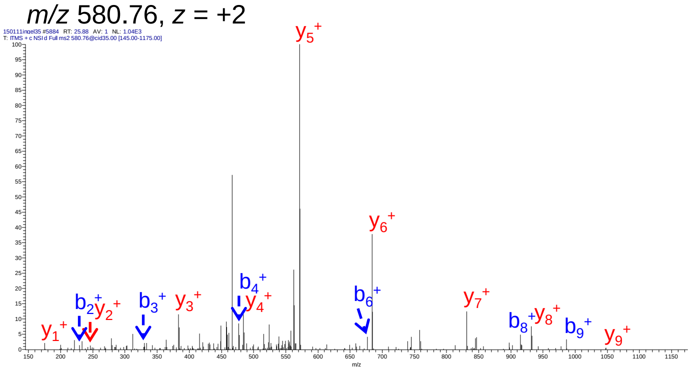

j

Tast2c 388-398 DISELNAFLCK

m/z 655.30, *z* = +2

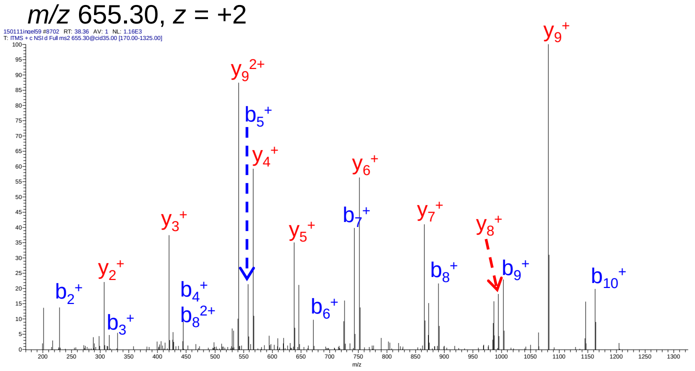

k

Tast2d 344-358 SPTLTFTGSLLEVLR

m/z 817.43, *z* = +2

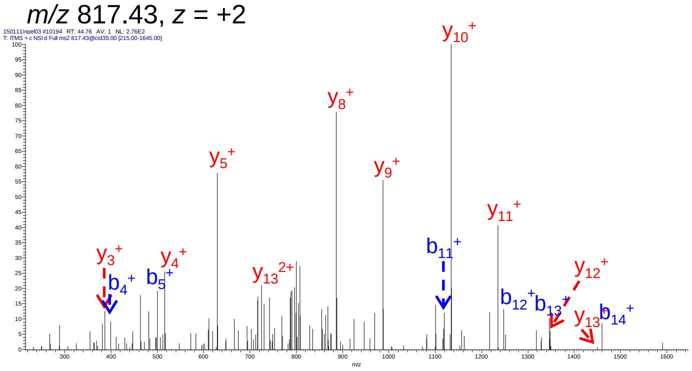

Fig. S3 Detection of Tast proteins in the sperm surface protein-enriched fraction (Nakazawa 2015) by mass spectrometry. a-e: The detected peptides at the ions score cut-off value of 20 are highlighted with orange bold font. One of the unique peptides shown in g-k are underscored. f: The sum of the protein scores of each protein from three independent preperations and scans. g-k: The product ion spectrum and annotation of one of the detected unique peptides for each Tast protein is shown.

Fig. S4

a

xxxxxxx...: intron/non-transcriptional region

XXXXXX...: exon

ATG: first ATG

left spacer right : target

C9.806 (*Tast1*) exons 1 and 2

gttgctacaacgtatcaccagacatgtatacggttaacacaattttaccagaacctgtc

→ C9.806exon1 primer

caattttatttatgtttttcaacattttgcattaagtttaattttttttttttaaattta

acatatttatgttcacaaattttggcgggaaattttggatcatcgtttaaaattcagac

exon 1

ATAATTTTTTACGATTGTAAAA**ATG**GAGGAACTGTATGTTAGCACTGGCCCTGCTCCGGA

C9.806-1

C9.806-2

AGTTGGGGAAAAGGAAAAACCTCGTCATCATCGCAAGCAGGCAGAAGCTGGCGGGGGTGG

C9.806-3

TAAGTCTGTGTTACGTAATATATTTGATTAgaaacaaaaaacaacaaccaccaacaaagt

aacatacttgaaaactcctaaactggcacgaggtgtatatgaagtattgaagtgttatac

actgttgtttttcagccacagagataaagttgcattttattcattcaatttatgtttatg

← C9.806e1r primer

tttagcctcaaacactaacagctgagcaaatacacacaaactagttaacaataagtgtgt

exon 2

ataatttttatttgtgtttactattttataccacattcaatttttttagTTCAAGGATCT

AAAAAACGTCCAACTAAGAAAGATGATAAAACTACTGTTAAAATAGCAATGGTTGGAATA

C9.806-4

CTTGTTGTTGGAGTAATAGCTCTCATTCTGGCTATTGTATTAGgtgagtgttttaaacgt

taaagtgtacgttatattatcatgtaaatggatgaaagcagttaataatggaaaaattgg

cacagtaattttagaggatattgatagtcttgatagaagacatgggtaaagggggcttg

ggtccaattttataaacatgggactatacaaagaagacggaaattttttccgctagagg

← C9.806e2r primer

cggtaattatttaaattaaaaaatacggaaaaacgaacaaaacagcactacaaagtgtta

ataagtgtaaaacaaattaattaaataataaaaatacctttctaaatacataaaacacta

Fig. S4 Continued

b

C1.533 (*Tast2a*) exon 1

aataacagcagcggcgagccctgaacctagattagaggcgcgccaaccactctgccacca

aacttaacaagatgcaaagttttattgattctcttttcttttatcaagttgtataccatt

C1.533exon1 primer →

exon 1

GACAAACTAGATTTGTAACAACAACCGCTTTTCAAAATTGCATAACTTTAAAATCTCTT

TATCACATTTTACATA**ATG**TTGGATACTTGTCTGGAACCATCTGGTGCACATAGCTCAAA

C1.533-1 C1.533-2

AGATGGCCAGCCAAGAAAAGGAAAGACAAAACAAGCCCAAACTGGAGgtagtgtactaag

ttcttgctatttaattttataaagtaatatatgcaaacatttaaaagctataaatataag

ggtgtacagtttttttacccaaaacaccaaagtagggcgatggaaaattattgatgttga

← C1.533e1r primer

gttatgtgtattttgaaaagtgtacattagtgtataacagactatagcactacttatgt

ttaatttgtaccaaacaacttaaaattgcactacagcaggtatttaaacccaagagagtt

c

C1.332 (*Tast2d*) exon 1

ttagttttaaaacacgtgatttgaacgttgtacatctggtctttgcgttcttattctgtg

C1.332e1r primer →

aacttattttgtaaaaaaaatttaattttgttttgtctgaaacgtgttattttgttgcag

exon 1

AAGGTCTATAGTATTAGGCTAGTATATTGTGACAAAATATGTATGCAAAGTAATCTTAAT

TGAATTAACAAAATGGATTTTTGT**ATG**GAACCTTCTGGTGCACACAACTCTAAAGATGGT

C1.332-1 C1.332-2

CGTCCCAAACATGGAAAAACAAAACAAGCCAAAACTGGAGgtgatggttttataattgtc

tcatattatgtaatattgttatatagaaaatttgtgatatattataatgttccttatgt

agtttttgtgttaatgagaacatcctactggcaccgcggcaaagtggttagcgcacatgc

ctctaacccagcggtaattggttcaaggcttgtcactgctaccattgtggacgttgggca

← C1.332exon1 primer

agacacaaattgtacaaattagcatccacacataaatattaaaaaaattcccaaaaaata

Fig. S4 Continued

d

targeted sites

```
left                spacer          right
ACGATTGTAAAAATGGaggaactgtatgttagCACTGGCCCTGCTCCGG
```

ATG: first ATG

C9.806-1: 9/9 (100%)

```
          ACGATTGTAAAAATGGaggaactgtatgttagCACTGGCCCTGCTCCGG
exon1     TTGTAAAAATGGAGGAACTGTATGTTAGCACTGGCCCTG-CTCCGGAAGTTGGGGAAAAG
1-2       TTGAAAAAATGGAGNAAC-----------ACTGCCCCCGGCTCCGGAAANTTGCNG----   -11
1-14      TTGTAAAAATGGAGGAAC-----------ACTGGCCCTG-CTCCGGAAGTTGGGGAAAA-
1-3       TGGNAAAAAGGGAGGANTT-----------NNGGCCCGG-GNCCGGAAGTTN--------   -12
1-4       TTGAAAAAATGGAGCAACT-----NCCCCACTGNCCCTG-CNCCGANAACTTGCN-----   -9+4
1-6       TTGTAAAAATGGANGAACC----GCCACCACCGCCCCNCCNCCCNCAAGN----------   -9+5
1-8       TTGTAAAAATGGAGGAACG---TGTTAGCACTGGCCCTG-CTCCGGAAGNTGGGGAAAA-   -3
1-11      TTGTAAAAATGGAGGAACT-----TTACCACTGGCCCTG-CTCCGGAAGTTGGGGAAAAN   -5
1-13      TTGTAAAAATGGAGGAACG-----TTAGCACTGGCCCTG-CTCCGGAAGTTGGGGAAAAG
1-15      TTGTAAAAATGGANNAACC----------CACTGNCCCCN-GCNCCGNNANNN--------   -9
```

C9.806-2: 13/13 (100%)

```
          GGAGGAACTGTATGTtagcactggccctgct---------------------CCGGAAGTTGGGGAAA
exon1     GGAGGAACTGTATGTTAGCACTGGCCCTGCT---------------------CCGGAAGT
2-1       GGANGANCTGTATGTTAGCAC------TGCT---------------------CCGGAAG-   -6
2-3       GGAGGAACTGTATGTTAGCNC------TGCN---------------------CCGGAAGT
2-5       GGAGGAACTGTATGTTAGCAC------TGCT---------------------CCGGAAGT
2-7       GGAGGAACTGTATGTTAGCAC------TGCT---------------------CCGGAAGT
2-12      GGAGGAACTGTATGTTAGCAC------TGCT---------------------CCGGAAGT
2-2       GGAGGAACTGTATGTTA--ACC-----TGCT---------------------CCGGANGT   -8
2-4       GGAGGAACTGTATGTTAGCACT-----TGCTCCGTTAGCACTTGCTCCTGCTCCGGAAGT   -5+21
2-6       GGAGGAACTGTATGTTAGCACC-----TGCT---------------------CCGGAAGT   -5
2-8       GGAGGAACTGTATGTTAGCACC-----TGCT---------------------CCGGAAGT
2-9       GGAGGAACTGTATGTTAGCACC-----TGCT---------------------CCGGAAGT
2-13      GGAGGAACTGTATGTTAGCACC-----TGCT---------------------CCGGAAGT
2-11      GGAGGAACTGTATGTTAGCACTG---CTGCT---------------------CCGGAAGT   -3
2-15      GGAGGAACTGTATGTTAGCACTGGC--TGCT---------------------CCGGAAGT   -2
```

C9.806-3: 3/3 (100%)

```
                  CATCATCGCAAGCAGgcagaagctggcgggggtgGTAAGTCTGTGTTACGT
exon1     AAAAACCTCGTCATCATCGCAAGCAGGCAGAAGCTGGCGGGGGTGGTAAGTCTGTGTTAC
3-3       AAAAACCCCGTCATCATNNCAAGCAGGCAAAAGCTGG----GGGGGTAAGTCTGTGTTAC   -4
3-10      AAAAACCTCNTCNTCATCGCAAGCAGGCAAAAGCTGG----GGTGGTAAGTCTGGGTTAC
3-15      AAAAACCTCGNCATCTTCGCAAGCAGGCAAAAG------GGGGGGGTNANNCTGTGTTAC   -6
```

C9.806-4: 16/16 (100%)

```
               CCAACTAAGAAAGATGataaaa----------------------ctactgttaAAATAGCAATGGTTGG
exon2     AACGTCCAACTAAGAAAGATGATAAAA----------------------CTACTGTTAAA
4-1       AACGTCCAACTAAGAAAGATGATAAA--------------------------CTGTTAAA   -4
4-2       AACGTCCAACTAAGAAAGATGATAAA--------------------------ATGTTAAA
4-3       AACGTCCAACTAAGAAAGATGATAAA--------------------------CTGTTAAA
4-9       AACGTCCAACTAAGAAAGATGATAAA--------------------------CTGTTAAA
4-12      AACGTCCAGCTAAGAAAGATGATAAA--------------------------CTGTTAAA
4-13      AACGTCCAACTAAGAAAGATGATAAA--------------------------CTGTTAAA
4-16      AACGTCCAACTAAGAAAGATGATAAA--------------------------CTGTTAAA
4-5       AACGTCCAACTAAGAAAGATGATAAC---------------------------TGTTAAA   -5
4-7       AACGTCCAACTAAGAAAGATGATAAC---------------------------TGTTAAA
4-4       AACGTCCAACTAAGAAAGANGAT--A--------------------------CTGTTAAA   -6
4-6       AACGTCCAACTAAGAAAGATGATAAAAAAAATAGCAATGGTTGGAATACTTGTTGTTAAA   -4+26
4-8       AACGTCCAACTAAGAAAGATGATAAAA------------------------------AAA   -8
4-10      AACGTCCAACTAAGAAAGATGA----------------------------------TAAA   -12
4-11      AACGTCCAACTAAGAAAGATGATAA---------------------------CTGTTAAA   -5
4-14      AACGTCCAACTAAGAAAGATGA------------------------------CTGTTAAA   -8
4-15      AACGTCCAACTAAGAAAGATGATA----------------------------CTGTTAAA   -6
```

Fig. S4 Continued

e

C1.533-1: 16/16 (100%)

```
                    AATGTTGGATACTTGTctggaacc---------a------tc------tggtGCACATAGCTCAAAAG
exon1           ACATAATGTTGGATACTTGTCTGGAACC---------A------TC------TGGTGCAC
1-2             ACATAATGTTGGATACTTGTCTG-------------------------------GTGCAC  -10
1-4             ACATAATGTTGGATACTTGTCTG-------------------------------GTGCAC
1-7             ACATAATGTTGNATACTTGTCTG-------------------------------GTGCAC
1-8             ACATAATGTTGGATACTTGTCTG-------------------------------GTACTC
1-12            ACATAATGTTGGATACTTGTCTG-------------------------------GTGCAC
1-13            ACATAATGTTGGATACTTGTCTG-------------------------------GTGCAC
1-14            ACATAATGTTGGATACTTGTCTG-------------------------------GTGCAC
1-15            ACATAATGTTGGATACTTGTCTG-------------------------------GTGCAC
1-16            ACATAATGTTGGATACTTGTCTG-------------------------------GTGCAC
1-1             ACATAATGTTGGATACTTGTCTGGAAT------------------------CTGNTGCTC  -3
1-3             ACATAATGTTGGATACTTGTCTGGAACC---------ACATAGCTCATAAGATGGTGCAC  -2+14
1-5             ACATAATGTTGGATACTTGTCTGGA---------------------------------AT  -12
1-6             ACATAATGTTGGATACTTGTCTGGAATC------------------------TGGTGCAC  -4
1-9             ACATAATGTTGGATACTTGTCTGGAAC-------------------------TGGTGCAC  -5
1-10            ACATAATGTTGGATACTGGT------------------------------------GCAC  -15
1-11            ACATAATGTTGGATACTTGTCTGAAACATAATGTTGGATACTTGTCTGGATCTGGTGCAC  -8+19
```

C1.533-2: 16/16 (100%)

```
                     ACTTGTCTGGAACCATctggt------gca------------------------------catagctCAAAAGATGGCCAGCCA
exon1       ATGTTGGATACTTGTCTGGAACCATCTGGT------GCA------------------------------CATAGCTCAAAAGATGGCCAGCCAAGAAAAG
2-1         ATGTTGGATACTTGTCTGGAACCATC----------------------------------------------AGCTCAAAAGATGGCCAGCCAAGAAAAG  -10
2-2         ATGTTGGATACTTGTCTGGAACCATCTGGT------------------------------------------AGCTCAAAAGATGGCCAGCCAAGAAAAG  -6
2-5         ATGTTGGATACTTGTCTGGAACCATCTGGT------------------------------------------AGCTCAAAAGATGGCCAGCCAAGAAAAG
2-7         ATGTTGGATACTTGTCTGGAACCATCTGGT------------------------------------------AGCTCAAAAGATGGCCAGCCAAGAAAAG
2-13        ATGTTGGATACTTGTCTGGAACCATCTGGT------------------------------------------AGCTCAAAAGATGGCCAGCCAAGAAAAG
2-3         ATGTTGGATACTTGTCTGGAACCAT-----------------------------------------------AGCTCAAAAGATGGCCAGCCAAGAAAAG  -11
2-6         ATGTTGGATACTTGTCTGGAACCAT-----------------------------------------------AGCTCAAAAGATGGCCAGCCAAGAAAAG
2-4         ATGTTGGATACTTGTCTGGAACCATCTGGT-------------------------------------------GCTCAAAAGATGGCCAGCCAAGAAAAG  -7
2-10        ATGTTGGATACTTGTCTGGAACCATCTGGT-------------------------------------------GCTCAAAAGATGGCCAGCCAANN----
2-8         ATGTTGGATACTTGTCTGGAACCATCTGGTG---------------------------------------GTAGCTCAAAAGATGGCCAGCCAAGAAAAG  -4+1
2-9         ATGTTGGATACTTGTCTGGAACCATCTGGTG---------------------------------------GTAGCTCAAAAGATGGCCAGCCAAGAAAAG
2-11        ATGTTGGATACTTGTCTGGAACCATCTGGTCATTTTACATAATGTTGGATACTTGTCAGAACCATCTATCATAGCTCAAAAGATGGCCAGCCAAGAAAAG  -3+29
2-12        ATGTTGGATACTTGTCTGGAACCATCTGGTG------------------------------------------GCTCAAAAGATGGCCAGCCAAGAAAAG  -6
2-14        ATGTTGGATACTTGTCTGGAACCATCT---------------------------------------------AGCTCAAAAGATGGCCAGCCAAGAAAAG  -9
2-15        ATGTTGGATACTTGTCTGGAACCATCTGGT-------------------------------------------GCTCAAAAGATGGCCAGCCAAGAAAAG  -12
2-16        ATGTTGGATACTTGTCTGGAACCATCTGGTGAT---------------------------ATATTTTATTGTAGCTCAAAAGATGGCCAGCCAAGAAAAG  -4+13
```

f

C1.332-1: 10/14 (71.4%)

```
      TTTTGTATGGAACCTTctggtgcaca--------------caactcTAAAGAT--GGTCGTCCC
exon1           ACCTTCTGGTGCACA--------------CAACTCTAAAGAT--GGTCG-TCCCAAACAT
1-6             ACCTTCTGGTGCACA--------------CAACTCTAAAGAT--GGTCG-TCCCAAACAT
1-9             ACCTTCTGGTGCACA--------------CAACTCTAAAGAT--GGTCG-TCCCAAACAT
1-12            ACCTTCTGGTGCACA--------------CAACTCTAAAGNATGGNNCG-TCCCAAACNT
1-16            ACCTTCTGGTGTAAAA-------------CAACTCTAAAGAT--GGTCG-TCCCAAACAT
1-1             ACCTTCTGGTGCT-------------------CTCTAAAGAT--GGTCG-TCCCAAACAT  -5
1-2             ACCCCCTT-----CA--------------CAANTCTNAANAT--GGTCT-NCCCANACAT  -5
1-3             ACCTTCTGGTGGAACC------------TCAACTCTAAAGAT--GGTCGATCCCAAAC--  +2
1-4             ACCTTCTGGTG------------------------TAAAGAT--GGTCG-TCCCAAACAT  -9
1-5             ACCTTCTGGTGA------------------ATCTCTA-----------------------  -9?
1-7             ACCTTCTGGTGCTCTAAAG-----------AACTCTAAAGAT--GGTCG-TCCCAAACAT  -4+7
1-8             ACCTTC------------------------AACTCTAAAGAT--GGTCG-TCCCAAACAT  -10
1-11            ACCTTCTA---------------------CAACTCTAAAGAT--GGTCG-TCCCAAACAT  -7
1-14            ACCTTCTGGTGCTTGTATGGAACCTTCTTCAACTCTAAAGAT--GGTCG-TCCCAAACAT  -3+17
1-15            ACCTTCTGGTGC------------------AACTCTAAAGAA--A---------AAAAAT  -4
```

C1.332-2: 14/14 (100%)

```
                                          ATGGAACCTTCTGGTGcacacaactctaaaGATGGTCGTCCCAAAC
exon1           TTGAATTAACAAAA-TGGATTTTTGTATGGAACCTTCTGGTGCACACAACTCTAAAGATG
2-1             TTGAATTAACAAAA-TGGATTTTTGTATGGAACCTTCTGGTGCACAC--------AGATG  -8
2-6             TTGAATTAACAAAA-TGGATTTTTGTATGGAACCTTCTGGTGCACAC--------AGATG
2-2             TTGAATTAACAAAA-TGGATTTTTGTATGGAACCTTCTGGTGCACAC-----TAAAGATG  -5
2-7             TTGAATTAACAAAA-TGGATTTTTGTATGGAACCTTCTGGTGCACAC-----TAAAGATG
2-8             TTGAATTAACAAAA-TGGATTTTTGTATGGAACCTTCTGGTGCACAC-----TAAAGATG
2-14            TTGAATTAACAAAA-TGGATTTTTGTACGGAACCTTCTGGTGCACAC-----TAAAGATG
2-3             TTGAATTAACAAAA-TGGATTTTTGTGTGGAACCCTCTGGTGGTCTA-------AAGATG  -7
2-9             TTGAATTAACAAAA-TGGATTTTTGTACGGAACCTTCTGGTGCACTA-------AAGATG
2-11            TTGAATTAACAAAA-TGAATTTTTGTATGGAACCTTCTGGTGCACTA-------AAGATG
2-12            TTGAATTAACAAAA-TGAATTTTTGTATGGAACCTTCTGGTGCACAC-------AAGATG
2-4             TTGAATTAACAAAA-TGGATTTTTGTATGGAACCTTCTGGTGCACAC----------ATG  -10
2-13            TTGAATTAACAAAAATGGATTTTTGTATGGAACCTTCTGGNGCACACTT----AAANATG  -10+6
2-15            TTGAATTAACAAAA-TGGATTTTTGTATGGAACCTTCTGGTGCACACA------AAGATG  -6
2-16            TTGAATTAACAAAA-TGGATTTTTGTACGGAACCTTCTGGTGCACACTC---TAAAGATG  -3
```

Fig. S4
Designs of TALEN pairs targeting Tast genes.
a-c. The targets of the left and right TALENs and the primers for genotyping are shown in the genome sequences.
d-f. The genotypes observed in the TALEN-injected tailbud larvae and the efficiency of mutagenesis caused by TALEN pairs.

Fig. S5

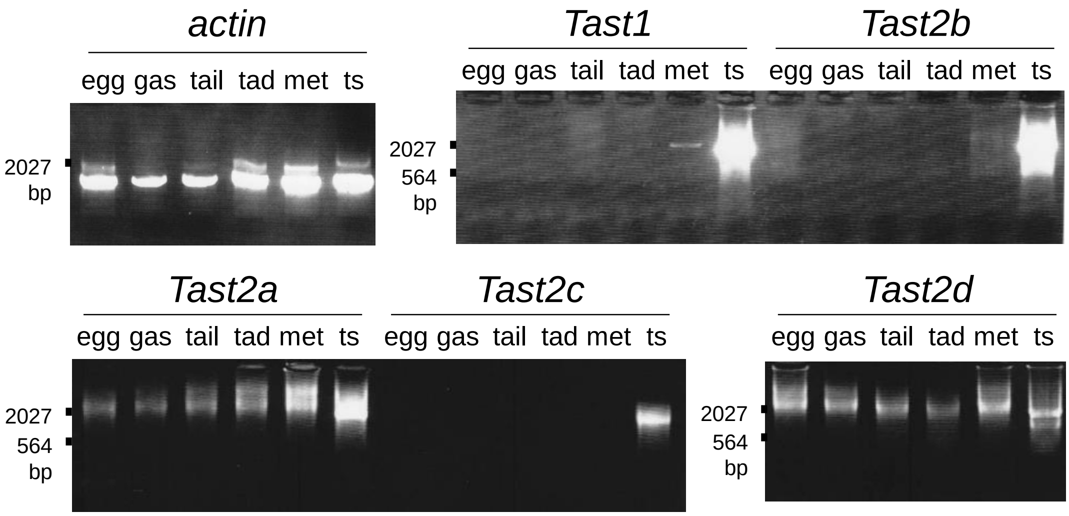

egg: unfertilized eggs (0 hpf)
gas: gastrula (3 hpf)
tail: tailbud larvae (8 hpf)
tad: tadpole larvae (14 hpf)
met: metamorphic larvae after tail contraction (44 hpf)
ts: adult testes

Fig. S5 At least three *Tast* genes were expressed at the developmental stages.

Fig. S6

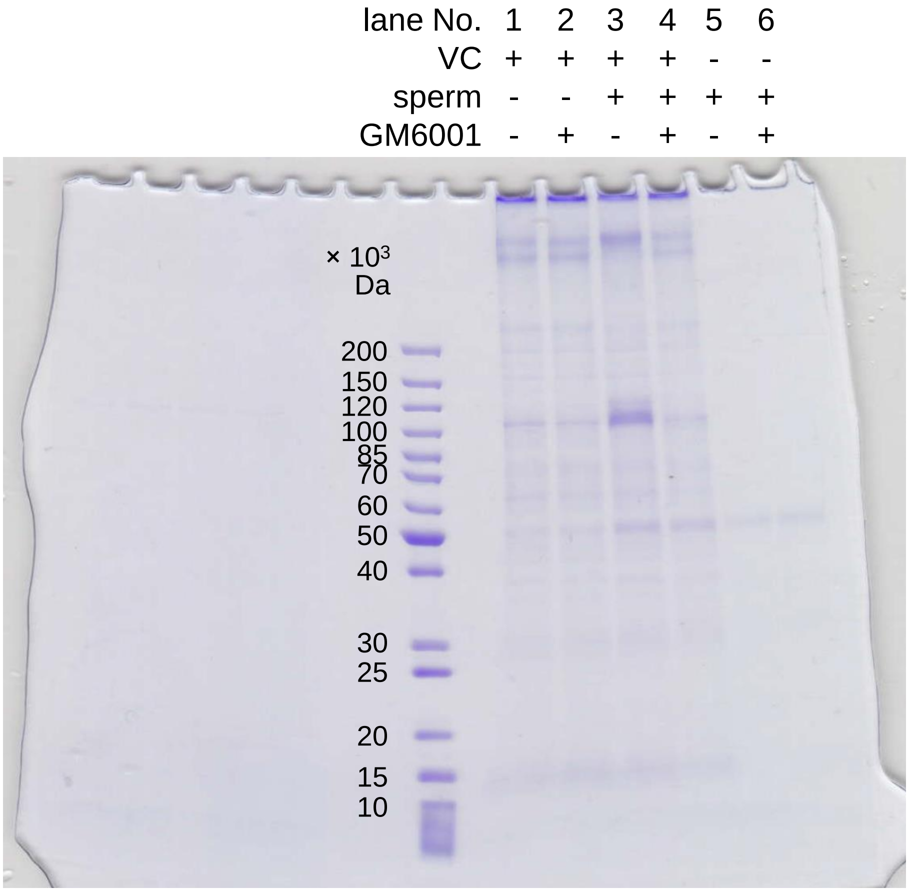

Fig. S6 The whole image of the gel shown in Figure 2.

Supplementary Table 1 The primers for amplification of the full coding sequences of *Tast* genes.

Target	Primer name	Sequence	Restriction site
Tast1	C9.806for	TTGGTACCATTGTAAAAATGGAGGAAC	*Kpn*I
Tast1	C9.806rev	TGTGGATCCACAAGATAACAATAAGC	*Bam*HI
Tast2b	C1.618for	TAGGTACCAAAATAAAATCTGAAATGG	*Kpn*I
Tast2b	C1.618rev	AATGGATCCAATGAAATGTCTTTTATG	*Bam*HI
Tast2a	C1.533for	TCGGTACCACATTTTACATAATGTTG	*Kpn*I
Tast2a	C1.533rev	CTGGGATCCTAGATGTTAAACTGGTT	*Bam*HI
Tast2c	C1.493for2	AAAggtacCGAATTATTCAAGTGGCC	*Kpn*I
Tast2c	C1.493rev	TTGGGATCCATTTTTGTTGATCAC	*Bam*HI
Tast2d	C1.332for	GAGATATCAAAATGGATTTTTGTATGG	*Eco*RV
Tast2d	C1.332rev	AACGGATCCAATTTCATTAATATAAC	*Bam*HI

Supplementary Table 2 The primers designed for 5'-RACE of *Tast2c*.

Target	Primer name	Sequence	Restriction site
BglII site + oligo (dC)	BglII-(dC)10	AAGATCTCCCCCCCCCC	*Bgl*II
C1.493 GSP for 5'-RACE	C1.493RACE1	ctcggatCCTTTAACATAGAGAGCAC	*Bam*HI

Supplementary Table 3. The primers used in confirmation of the sequences of cloned *Tast* genes.

Target	Primer name	Sequence	Restriction site
pBluescript universal primer-binding site	M13-20	CGACGTTGTAAAACGACGGCCAGT	-
pBluescript universal primer-binding site	T3 promoter	ATTAACCCTCACTAAAGGGAA	-
Tast1	C9.806seq1	GGGATTGGTTGTTCAAAAGC	-
Tast1	C9.806seq2	TGTGTTCCCTTCACATTTGC	-
Tast2b	C1.618seq1	TATGGGTGCGATTATGTCGG	-
Tast2b	C1.618seq2	ACTGTGACCTTCTAGGACAG	-
Tast2a	C1.533seq1	GCTGTTCTTCTTCCATCGGG	-
Tast2a	C1.533seq2	TGTTCCCCAAGCAATTTGAG	-
Tast2c	C1.493seq1	AAGATATTTCTGAGCTGAATGC	-
Tast2c	C1.493seq2	CCACATGTAGTTGTGCATGC	-
Tast2d	C1.332seq1	GAATTTCTTGACAAATACGGG	-
Tast2d	C1.332seq2	GCCATCACACTTCCATGATG	-

Supplementary Table 4. The primers for PCR amplification and genotyping of TALEN-targeted sequences.

Target	Primer name	Sequence	Restriction site
Tast1 exon 1	C9.806exon1	cagacatgtatacggttaac	-
Tast1 exons 1~2	C9.806e2r	ccgtcttctttgtatagtcc	-
Tast2a exon 1	C1.533exon1	ctgccaccaaacttaacaag	-
Tast2a exon 1	C1.533e1r	catcaataattttccatcgcc	-
Tast2d exon 1	C1.332e1f	ctttgcgttcttattctgtg	-
Tast2d exon 1	C1.331exon1	acaagccttgaaccaattac	-
